# Prognostic Significance of the Combined Albumin-To-Alkaline Phosphatase Ratio (AAPR) and Haemoglobin–Albumin–Lymphocyte–Platelet (HALP) Score in Patients with Metastatic Renal Cell Carcinoma Treated by Targeted Therapy: A New Prognostic Combined Risk Scoring

**DOI:** 10.3390/jcm14051742

**Published:** 2025-03-05

**Authors:** Tolga Köşeci, Mustafa Seyyar, Mehmet Mutlu Kıdı, Sedat Biter, Kadir Eser, Umut Kefeli, Erdinç Nayır, Berna Bozkurt Duman, Burak Mete, Hakan Demirhindi, Timuçin Çil

**Affiliations:** 1 Department of Medical Oncology, Faculty of Medicine, Çukurova University, 01330 Adana, Türkiye; mkidi@cu.edu.tr (M.M.K.); sbiter@cu.edu.tr (S.B.); 2 Department of Medical Oncology, Gaziantep City Hospital, 27470 Gaziantep, Türkiye; mustafaseyyar@saglik.gov.tr; 3 Department of Oncology, Mersin University Hospital, 33240 Mersin, Türkiye; kadireser@mersin.edu.tr; 4 Department of Medical Oncology, Faculty of Medicine, Kocaeli University, 41001 Kocaeli, Türkiye; umut.kefeli@kocaeli.edu.tr; 5 Department of Medical Oncology, Mersin Medical Park Hospital, 33200 Mersin, Türkiye; erdinc.nayir@toros.edu.tr; 6 Department of Medical Oncology, Adana City Training and Research Hospital, University of Health Sciences, 01370 Adana, Türkiye; bernabozkurt.duman@sbu.edu.tr (B.B.D.); timucin.cil@sbu.edu.tr (T.Ç.); 7 Department of Public Health, Faculty of Medicine, Çukurova University, 01330 Adana, Türkiye; bmete@cu.edu.tr

**Keywords:** renal cell carcinoma, tyrosine kinase inhibitors, AAPR, HALP, prognosis

## Abstract

**Background/Objectives:** Renal cell carcinoma (RCC) accounts for 2–3% of all cancers, with approximately 25% of patients being detected at the metastatic stage. This study aimed to determine the prognostic significance of co-evaluating two risk parameters: one, the HALP score based on haemoglobin, albumin, lymphocyte, and platelet counts, and the other, albumin-to-alkaline phosphatase ratio (AAPR) in patients with metastatic RCC treated with targeted therapy. **Methods:** This retrospective cohort study included 147 patients with metastatic RCC. The HALP score and AAPR values were calculated from pre-treatment blood test results, and followingly, the cut-off value was determined by using ROC analysis. Patients were categorised into three groups with a low, moderate or high combined risk score based on this cut-off value. The effect of these risk groups on survival was evaluated. **Results:** The mean age of patients was 64.1 ± 11.9. The median follow-up time was 38.3 months, and the mortality rate was 53.7% in all groups. Kaplan–Meier survival analysis showed a statistically significant difference between the combined scores of the risk groups: the median survival time was 51.6 months in the low-risk group, 20.7 months in the medium-risk group, and 10.4 months in the high-risk group (*p* < 0.001), with 5-year survival rates being 38.8% in the low-risk group, 30% in the intermediate-risk group, and 19% in the high-risk group. When compared to the low-risk group, Cox regression analysis revealed that the mortality risk, i.e., HR (hazard ratio), was 2.42 times higher in the intermediate-risk group and 3.76 times higher in the high-risk group. A nephrectomy operation decreased the mortality risk (HR = 0.24) by 4.16 times. **Conclusions:** This new combined risk scoring, obtained from co-evaluating the HALP score and AAPR, was found to be an independent prognostic factor in metastatic RCC patients. This combined risk scoring is expected to help clinicians in treatment decisions.

## 1. Introduction

Renal cell carcinoma (RCC) constitutes approximately 2–3% of all malignancies, with a stable prevalence over the years ranging between 2 and 3% in 2005 and 2.2% in 2020 [1]. Approximately 25% of patients diagnosed with RCC are metastatic at the time of diagnosis [2]. Clear-cell renal cell carcinoma is reported as the leading histopathological type, observed in approximately 70% of all cases, followed by papillary, chromophobe, and collecting duct tumours [3]. Recurrence is observed in 30% of patients after surgery [4]. The prognosis of metastatic renal cell carcinoma (mRCC) is poor, but a median overall survival of 26 to 28 months can be achieved after introducing tyrosine kinase inhibitors in the therapy protocol [2,5,6]. Moreover, recent studies reported effective immune checkpoint inhibitor treatments, referred to as “target therapy” [7,8]. For mRCC patients undergoing targeted therapy, many validated prognostic models are frequently employed to assess prognosis and support the best possible management decisions [9]. Dietary and immunological status are currently considered significant predictive factors, but they are not accounted for in these models.

Haemoglobin, albumin, lymphocyte, neutrophil, and platelet values that can be quickly determined in blood samples provide information about patients’ nutritional and inflammatory conditions, as emphasised in many studies on renal cell carcinoma [10,11]. However, compared to a single index, better success in predicting prognosis was achieved when these markers were evaluated in combination. For this purpose, the lymphocyte-to-monocyte ratio, neutrophil-to-lymphocyte ratio, and prognostic nutritional index have been highlighted in studies of renal cell carcinoma [12,13,14].

Cancer is a complex disease that can affect various parts of the human body, in addition to human metabolism, the musculoskeletal system, and nutritional status [15,16]. Many studies have shown that immune response and tumour-related nutritional consumption contribute to disease progression. The albumin-to-alkaline phosphatase ratio (AAPR), which is obtained by dividing the serum albumin level by that of serum alkaline phosphatase, was first examined in patients with hepatocellular carcinoma in 2015 [17]. Consequently, research conducted on a variety of malignancies, like breast cancer, lung cancer, and nasopharyngeal cancer, has demonstrated the predictive significance of AAPR [18,19,20]. There is no study about the AAPR index in patients with mRCC who were treated with tyrosine kinase inhibitors. A unique combination of haemoglobin, albumin, lymphocytes, and platelets (HALP) was initially examined in gastric cancer in 2015, identifying the HALP score as being strongly associated with the clinicopathological aspects of the disease [21]. There is only one study on the HALP index in patients receiving tyrosine kinase inhibitor therapy for mRCC.

Thus, we aimed to analyse both HALP and AAPR in combination in patients with mRCC, and to simultaneously investigate both indexes in these patients. This will be the first study that evaluates the “combined risk scoring”, as far as we know from the literature review.

## 2. Materials and Methods

### 2.1. Research Type and Selection of Participants

**Figure 1:**
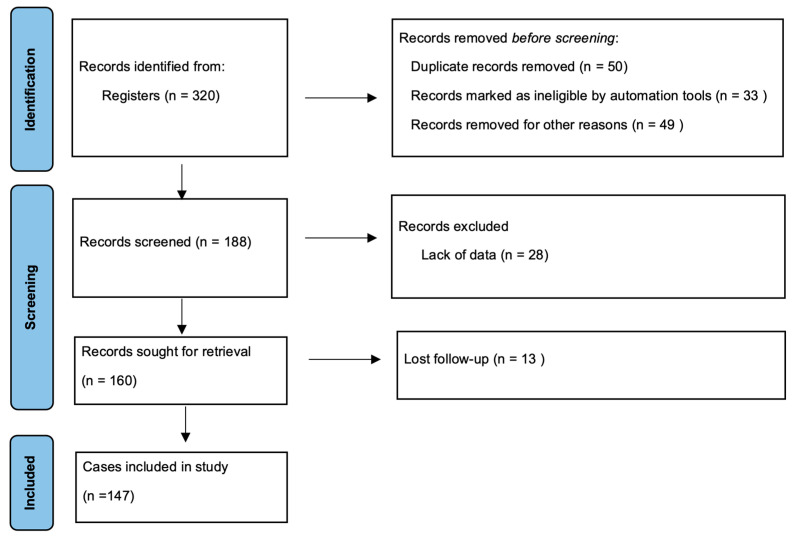
Flow chart for the selection of patients.

This retrospective cohort study was conducted on patients with a diagnosis of RCC who received targeted treatment with tyrosine kinase inhibitors (50 mg/day sunitinib for 4 weeks followed by a pause of 2 weeks and 800 mg/day pazopanib) between 2011 and 2021 in the oncology departments of Adana City Hospital and Kocaeli University’s Faculty of Medicine. This study was approved by the local ethics committee of Adana City Hospital (1204/30 December 2020). Based on the results of a reference study [22], the minimum number of samples planned to be included in the study was determined as 117, with an effect size of 0.30, a 95% confidence interval and 90% power, calculated according to the chi-square test. The sample size was assessed by using the G*power 3.1.9.4 programme. During the study period, the files of 320 patients diagnosed with RCC were scanned. A total of 147 patients subject to target therapy with tyrosine kinase inhibitors were included in the study according to the inclusion and exclusion criteria. Patients younger than the age of 18 years, those with non-metastatic RCC, and those with secondary malignancy or autoimmune disease, heart failure, active inflammation, and inflammatory disease were excluded from the study, in addition to patients lacking sufficient clinical information. The selection procedure is presented in the flow chart in Figure 1.

### 2.2. Patients’ Data and Tumour Staging

Clinicopathological characteristics and laboratory data were collected. For tumour staging before the first cycle, either positron emission tomography (PET) or thoracic–abdominal–pelvic computed tomography (CT) were performed based on the doctor’s preference. The patient data were reviewed retrospectively. Patients’ age, sex, tumour histology, tumour grade, Memorial Sloan Kettering Cancer Center (MSKCC) prognostic model risk group, calcium, lactate dehydrogenase, haemoglobin, albumin, lymphocyte, platelet values, nephrectomy status, death time, metastasis side, and tumour location were collected. The patients underwent PET and CT examination to ascertain the extent of the metastases. The patients were evaluated with CT or PET-CT throughout the therapy period. The Declaration of Helsinki’s ethical guidelines for medical research involving human participants were followed in collecting patient data. Despite obtaining the ethics committee’s approval, we did not ask patients for their informed consent because our study was retrospective, and only hospital records were anonymously used.

### 2.3. Calculation of HALP-AAPR Risk Index Couple

Before starting targeted therapy, patients’ alkaline phosphatase, albumin and haemoglobin levels, and platelets and lymphocyte counts were determined, followed by the calculation of the HALP score [21] and AAPR [17], as shown below:

$$HALP\;score=\frac{{Haemoglobin\;\left(g/L\right)\times Albumin\;\left(g/L\right)\times Lymphocyte\;count\;(L}^{-1})}{Platelet\;count\;{(L}^{-1})}$$

$$AAPR=\frac{Albumin\;(g/L)}{Alkaline\;phosphatase\;(IU/L)}$$

### 2.4. ROC Analyses and Cut-Offs

The cut-off value was calculated based on receiver operating characteristics (ROC) analysis. The cut-off was found to be 29.4 (AUC = 0.602, *p* = 0.034) for the HALP score and 0.40 (AUC = 0.769, *p* < 0.001) for the AAPR. The patients were grouped based on the cut-off value according to the ROC analysis results: “low-HALP group” if the AUC was ≤29.4 and “high-HALP group” if the AUC was >29.4, while they were “low-AAPR group” if the AUC was ≤0.40; and “high-AAPR group” if it was >0.40. We combined these two inflammatory indexes and created combined risk scoring (CRS) groups. Patients in both the high-AAPR and the high-HALP groups (AUC > 0.40 and >29.4, respectively) were classified as the “low combined risk scoring group (low CRS)”, and patients in either the low-AAPR or the low-HALP group were classified as the “moderate combined risk group (moderate CRS)”, and patients in both low-AAPR and the low-HALP group (AUC ≤ 0.40 and ≤29.4, respectively) were classified as the “high combined risk group (high CRS)” (Table 1).

### 2.5. Statistical Analysis

IBM SPSS, version 20.0 (IBM Corp., Armonk, NY, USA) and JAMOVI 2.6.17 (URL: https://www.jamovi.org accessed on 1 October 2024) were used for data analysis. The variables were investigated using probability plots and analytical methods (Kolmogorov–Smirnov test) to check the normality distribution. The relationship between the clinicopathological data and the inflammatory indexes (AAPR and HALP) was examined using the chi-square or Fisher’s exact tests. As the patients’ ages were normally distributed, an independent Student’s *t*-test or one-way ANOVA test was applied. However, as the tumour diameters were not normally distributed, Kruskal–Wallis tests were conducted. The capacity of AAPR and HALP values in predicting the presence of mortality was analysed using the ROC curve analysis. Overall survival (OS) was estimated using the Kaplan–Meier survival technique, and the relationship between OS and patient-related clinical parameters was examined using the log-rank test. OS was defined as the time until death that occurred following the initiation of tyrosine kinase inhibitor therapy. Data were censored if the patients were still alive at the time of the last clinical evaluation. Using a Cox proportional hazards model, the univariate analysis of the clinicopathological variables, AAPR, and HALP was carried out to yield hazard ratios (HRs) and 95% confidence intervals (CI). A *p*-value < 0.05 was considered statistically significant.

## 3. Results

The mean age of the patients was 64.4 years, with 77.6% (*n* = 114) male and 22.4% female (*n* = 33). Based on patients’ histology, clear cell carcinoma was diagnosed in 85.7%. The MSKCC risk grouping showed that 17.7% of the cohort had a favourable prognosis, 60.5% had an intermediate prognosis, and 21.8% had a poor prognosis. The Fuhrman grade was found as I-II in 39.5% (*n* = 58) of the patients and III-IV in 45.6% (*n* = 57). The median tumour diameter was 72 mm, and nephrectomy was present in 64.6% of the patients. We observed that 95 patients had lung metastasis, 17 patients had liver metastasis, 27 patients had lymph node metastasis, and 44 patients had bone metastasis. The distribution of demographic and clinical characteristics of the patients according to risk groups is given in Table 2.

**Figure 2:**
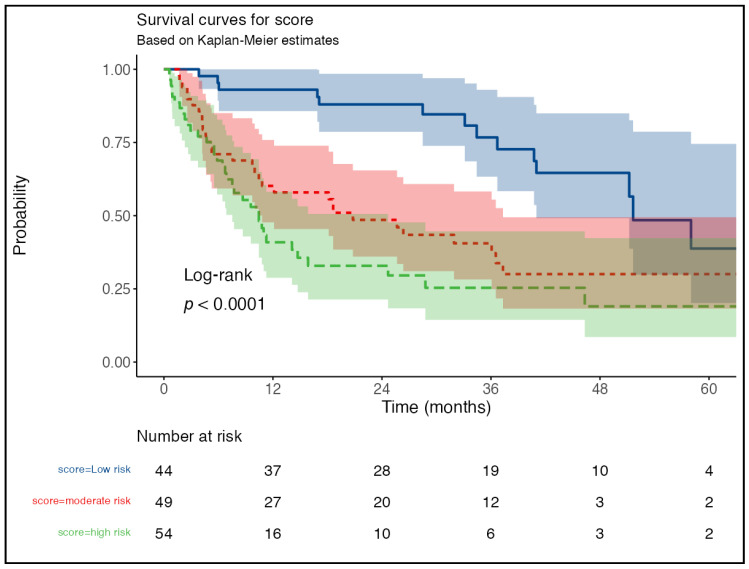
Survival curves according to combined risk scoring (Kaplan–Meier).

The HALP-AAPR Kaplan–Meier survival analysis of the effect of risk score groups on survival showed a statistically significant difference between the risk groups. The median OS time was 51.6 months in the low-CRS group, 20.7 months in the moderate-CRS group and 10.4 months in the high-CRS group, respectively (*p* < 0.001). When the survival times were compared between the groups, the median survival times of the patients in the low-CRS group were significantly longer than those in the moderate- and high-CRS groups, whereas there was no significant difference in the survival times between the moderate- and high-CRS groups (Table 3 and Figure 2).

When one-, three-, and five-year survival rates were evaluated according to the risk scoring groups, the survival rates of the patients in the low-risk CRS group were found to be 93.0%, 76.7%, and 38.8% at the end of the first, third, and fifth years, respectively. The survival rates of the patients in the moderate-risk CRS group were found to be 60.2%, 40.6%, and 30.0% at the end of the first, third, and fifth years, respectively. The survival rates of the patients in the high-risk CRS group were found to be 40.9%, 25.4%, and 19.0% at the end of the first, third, and fifth years, respectively (Table 4).

**Figure 3:**
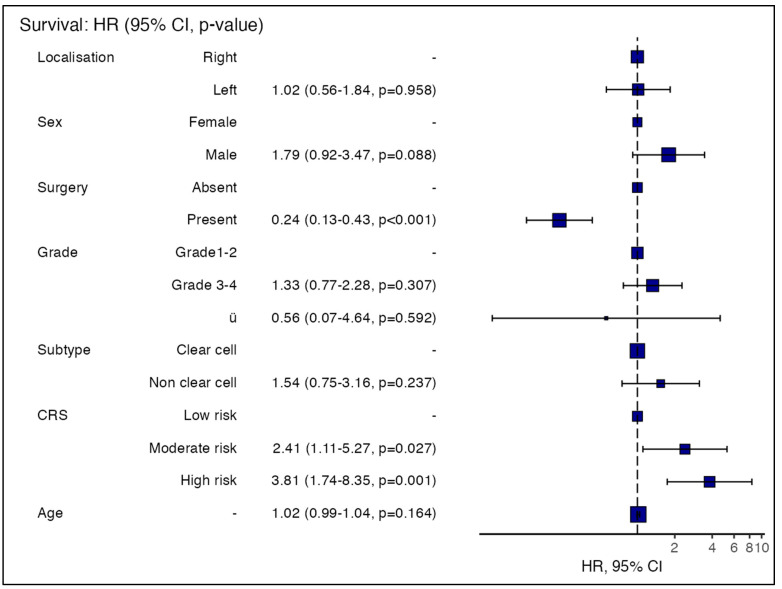
Hazards regression plot.

Cox regression analysis to predict mortality risk in RCC patients was found to be significant (*p* < 0.001). The independent variables of the model were age, sex, primary surgery status, tumour location, grade, histological subtype, and CRS. In univariate and multivariate analyses, it was observed that the patient’s history of nephrectomy and CRS were found to be effective indicators of mortality in the models. It was observed that the mortality risk in patients who underwent surgery during the follow-up period was 4.16 times lower (HR = 0.24). Moreover, compared to the low-risk group, the mortality risk was 2.42 times higher in the moderate-risk group and 3.76 times higher in the high-risk group (Table 5 and Figure 3).

## 4. Discussion

In this study, we investigated the prognostic significance of CRS, which was classified by using HALP score and AAPR values in patients with mRCC. Multivariate analyses showed that CRS was an independent prognostic factor in patients with mRCC. The risk of mortality (HR) during the follow-up period was 2.4-fold higher in the intermediate-CRS group (with a 5-year survival rate of 30%) and 3.76-fold higher in the high-CRS group (with a 5-year survival rate of 19%) when compared to the low-CRS group. The median survival time was 51.64 months for the low-risk group, 20.79 months for the intermediate-risk group, and 10.44 months for the high-risk group.

Prognostic factors are very important in selecting the treatment modality for patients with renal cell carcinoma. For this purpose, there are different prognostic risk score models defined, such as the Heng risk model. Some predictive factors used to show the prognosis of disease with mRCC were defined, such as tumour size, stage, and grade [23]. It was reported that inflammatory markers based on the haemogram parameters like neutrophil, lymphocyte, and platelet counts were associated with prognosis in RCC patients [11,12,13]. Combining inflammatory markers and albumin was declared to further emphasise the prognostic significance of these tests [24].

Nutritional status and tumour-related immune responses were associated with tumour development and progression [17]. It is unclear how the illness course and AAPR are related. Albumin was demonstrated to control DNA replication, immunological responses, and cell proliferation, in addition to its antioxidant properties that protect against carcinogens. Therefore, cancer patients with low albumin counts were linked to dietary deficiencies, inadequate anticancer responses, and reduced immunological responses [25]. Hepatitis, bile duct disorders, malnutrition, and bone disease may be linked to elevated alkaline phosphatase levels. Previous research showed that a high alkaline phosphatase level was associated with a poor prognosis. It was also shown that various cancer types present with high tumour burdens. Additionally, it was documented that alkaline phosphatase had a suppressive effect on the immunological system. Mori et al. reported a link between elevated alkaline phosphatase levels and micro-metastasis. This suggested that the prognosis would not be good for cancer patients with high alkaline phosphatase levels [26]. Alkaline phosphatase was expressed in cancer cells and linked to the growth of tumours [27]. The prognostic importance of AAPR was demonstrated by studies performed on different cancer types, such as hepatocellular and nasopharyngeal cancer [28,29]. AAPR was evaluated in renal cell carcinoma patients with or without metastasis [30,31]. Yoshino et al. examined the prognostic importance of AAPR in patients with mRCC who were treated with nivolumab in the second line. The AAPR value was an independent prognostic factor in their studies in multivariate analysis [31]. Overall survival was statistically significantly longer in patients with high AAPR than in those with low AAPR in our study. To the best of our knowledge, this is the first study to demonstrate that AAPR was significantly associated with the outcome of patients with renal cell carcinoma who were treated with targeted therapy.

Anaemia is seen in one-third of patients with cancer, and it is related to advanced-stage cancer [32]. It may occur due to the suppression of erythropoiesis by cytokines released due to the inflammatory process occurring in cancer patients. Advanced solid tumours frequently present with tumour hypoxia and poor tumour oxygenation, leading to increased resistance to therapy because of an imbalance between oxygen supply and consumption. Anaemia may aggravate tumour hypoxia and poor tumour oxygenation, and consequently decrease the effectiveness of therapy [33,34]. Anaemia may occur based on multifactorial causes in these patients [35]. Serum albumin is synthesised in the liver and is known as a negative acute-phase protein. Decreased serum albumin levels reflect malnutrition and inflammation [36]. Anaemia and hypoalbuminemia were associated with poor prognoses in different cancers, including RCC [37]. Further, platelets facilitate the tendency of cancer cells to metastasise. For example, platelets were shown to prevent cancer cells from being attacked by natural killer cells by building a barrier around the cancer cells [38]. Lymphocytes are an important part of the immune and inflammation systems. A low lymphocyte count indicates poor survival [39]. Low haemoglobin levels facilitate carcinogenesis by deepening hypoxia, while low albumin levels by decreasing antioxidant properties, low lymphocyte levels by decreasing immune response, high platelet levels by facilitating metastasis, and high alkaline phosphatase levels by contributing to tumour growth. Whereas increased albumin, lymphocyte, and haemoglobin counts were associated with good prognoses, high platelet counts were associated with poor prognoses.

The HALP score is calculated based on haemoglobin, lymphocyte, platelet, and albumin counts. Studies of many cancer types showed that patients with high HALP scores had better survival compared to those with low HALP scores [21,40]. The first study on the HALP score was performed by Peng et al. in patients with RCC in 2018. In their study, 90.2% of patients had clear cell carcinoma histology, while this histologic frequency was found to be 85.7% in our study. Peng et al. found a HALP score cut-off value of 31.2, similar to the cut-off of 29.4 that we found in our study. Tumour location was similar in both studies. Peng et al. first described the prognostic importance of the HALP score for survival among patients with renal cell carcinoma [41].

Chronic inflammation plays a critical role in tumour development, proliferation, and metastasis and is related to cancer death and recurrence in patients with cancer [42]. Clearly, cancer prognosis is not only related to tumour features. The immune response of the host has critical importance [43]. Nutritional deficiency is related to a poor anticancer response. We combined these two indicators to establish a novel prognostic score that had a superior predictive ability compared to either metric alone. In our study, the survival of patients with high AAPR and HALP scores was longer than that of patients with low AAPR and HALP scores. In daily practice, these simple, easy-to-calculate indices, which together yield CRS, can be used to determine the most efficient treatment modality among mRCC patients receiving tyrosine–kinase inhibitor therapy. There are studies on the HALP score in RCC patients receiving tyrosine kinase inhibitors but evaluating both scores in combination is a new approach and is expected to fill the gap in the literature regarding the selection of the most efficient modality for therapy. This constitutes one of the strengths of our study.

One of the limitations of the study is that it was retrospective. Secondly, we could not access some of the patients’ parameters, such as weight loss and medication. Previous studies had different cutoff values for the HALP score and AAPR. It is still not known which cut-off value is better for predicting overall survival. Our strength was that this was the only study in which both scores were used together.

## 5. Conclusions

The CRS (HALP-AAPR) that we developed indicated that both survival times and rates decreased with increasing risk scores, providing an important prognostic factor in mRCC patients receiving targeted therapy. The determination of CRS in the pre-treatment period may help clinicians decide on treatment options. There is a necessity for studies on a large-scale patient population to further validate the combination of HALP scores and AAPR for the prognosis of mRCC.

## Figures and Tables

**Table 1:** Assessment of combined risk scoring (CRS).

		HALP Score Classification	
		HALP ≤ 29.4	HALP > 29.4
AAPR classification	AAPR ≤ 0.40	High-CRS	Moderate-CRS
	AAPR > 0.40	Moderate-CRS	Low-CRS

AAPR, Albumin-to-alkaline phosphatase ratio; AUC, area under the curve; HALP, haemoglobin–albumin–lymphocyte–platelet; CRS, combined risk score.

**Table 2:** Comparison of sociodemographic and clinical characteristics according to the combined risk scoring groups.

		Combined Risk Scoring (CRS) Groups Mean ± S.D. or *n* (%)			
Variables		Low CRS *n* = 44	Moderate CRS *n* = 49	High CRS *n* = 54	*p*
Age (years)		63.4 ± 13.4	67.5 ± 11.2	62.3 ± 10.5	0.065
Sex	Male	36 (81.2)	38 (77.6)	40 (74.1)	0.659
	Female	8 (18.2)	11 (22.4)	14 (25.9)	
Histological subtype	Clear-cell carcinoma	43 (97.7)	43 (87.8)	40 (74.1)	0.003 *
	Non-clear-cell carcinoma	1 (2.3)	6 (12.2)	14 (25.9)	
Fuhrman	I–II	20 (51.3)	16 (42.1)	22 (45.8)	0.718
tumour grade	III–IV	19 (48.7)	22 (57.9)	26 (54.2)	
MSKCC subgroup	Favourable	17 (38.6)	6 (12.2)	3 (5.6)	<0.001 *
	Intermediate	25 (56.8)	32 (65.3)	32 (59.3)	
	Poor	2 (4.5)	11 (22.4)	19 (35.2)	
Nephrectomy	Present	7 (15.9)	22 (44.9)	22 (41.5)	0.006 *
	Absent	37 (84.1)	27 (55.1)	31 (58.5)	
Liver metastasis	Present	3 (6.8)	7 (14.3)	7 (13.0)	0.490
	Absent	41 (93.2)	42 (85.7)	47 (87.0)	
Lung	Present	30 (68.2)	35 (71.4)	30 (55.6)	0.204
metastasis	Absent	14 (31.8)	14 (38.6)	24 (44.4)	
Bone	Present	10 (22.7)	16 (32.7)	18 (33.3)	0.458
metastasis	Absent	34 (77.3)	33 (67.3)	36 (66.7)	
Lymph node	Present	3 (6.8)	8 (16.3)	16 (29.6)	0.013 *
metastasis	Absent	41 (93.2)	41 (83.7)	38 (70.4)	

Comparisons based on chi-squared tests (except “age” where Student’s *t*-test was used); *, statistical significance; CRS, combined risk scoring; MSKCC, Memorial Sloan Kettering Cancer, S.D., standard deviation; non-clear-cell carcinomas presented with papillary and chromophobe histopathological types.

**Table 3:** Survival time according to the combined risk scoring (CRS) groups.

	Mean Survival Time				Median Survival Time			
			95% CI				95% CI	
CRS Groups	Estimate	SE	Lower	Upper	Estimate (Months)	SE	Lower	Upper
Low risk	65.53	8.23	49.39	81.66	51.64 ^a,b^	4.58	42.65	60.63
Moderate risk	40.26	6.48	27.55	52.97	20.79 ^b,c^	4.99	11.00	30.58
High risk	29.41	6.59	16.48	42.33	10.44 ^a,c^	1.40	7.68	13.20
Overall	43.95	4.44	35.24	52.67	31.93	6.35	19.48	44.38

Statistics used: Kaplan–Meier survival technique; CRS, combined risk scoring; ^a,b,c^ pairwise comparisons with significance *p* < 0.001 for a, *p* < 0.001 for b, *p* = 0.185 for c; CI, confidence interval; SE, standard error.

**Table 4:** 1-year, 3-year, and 5-year survival rates according to the combined risk scoring (CRS) groups.

CRS Groups	Time (Month)	Number at Risk (*n*)	Number of Events (*n*)	Survival Rate % (95% CI)
Low risk	12	37	3	93.0 (85.7–100.0)
	36	19	5	76.7 (63.3–93.0)
	60	4	6	38.8 (20.2–74.5)
Moderate risk	12	27	19	60.2 (47.7–75.9)
	36	12	8	40.6 (28.2–58.3)
	60	2	3	30.0 (18.2–49.5)
High risk	12	16	28	40.9 (28.8–58.2)
	36	6	5	25.4 (14.4–44.6)
	60	2	1	19.0 (8.5–42.3)

Statistics used: Kaplan–Meier survival technique; CRS, combined risk groups; CI, confidence interval.

**Table 5:** Cox regression analysis for predicting mortality risk in renal cell carcinoma patients.

Independent Variables	Subgroups	*n* (%)	Univariate Hazard Ratio (95% CI, *p*)	Multivariate Hazard Ratio (95% CI, *p*)
Age	Mean (SD)	64.0 (11.6)	1.02 (0.99–1.04, *p* = 0.149)	1.02 (0.99–1.04, *p* = 0.165)
Sex	Female	30 (24.0)	-	-
	Male	95 (76.0)	1.26 (0.68–2.35, *p* = 0.458)	1.78 (0.91–3.46, *p* = 0.090)
Location	Right	70 (56.0)	-	-
	Left	55 (44.0)	0.60 (0.35–1.02, *p* = 0.058)	1.01 (0.56–1.83, *p* = 0.970)
Surgery	Absent	36 (28.8)	-	-
	Present	89 (71.2)	0.22 (0.13–0.38, *p* < 0.001 *)	0.24 (0.13–0.44, *p* < 0.001 *)
Histological subtype	Clear cell carcinoma	107 (85.6)	-	-
	Non clear cell carcinoma	18 (14.4)	1.75 (0.90–3.39, *p* = 0.097)	1.54 (0.75–3.15, *p* = 0.238)
Fuhrman tumour grade	Grade I–II	58 (46.4)	-	-
	Grade III–IV	67 (53.6)	1.00 (0.60–1.68, *p* = 0.987)	1.33 (0.77–2.28, *p* = 0.307)
Combined risk scoring group	Low risk	39 (31.2)	-	-
	Moderate risk	38 (30.4)	2.94 (1.38–6.25, *p* = 0.005 *)	2.42 (1.11–5.27, *p* = 0.026 *)
	High risk	48 (38.4)	4.37 (2.10–9.07, *p* < 0.001 *)	3.76 (1.72–8.23, *p* = 0.001 *)

Statistics used: Cox regression analysis; *, statistical significance; CI, confidence interval; SD, standard deviation. The power (1 − β) of the model was calculated as 91% by “a posteriori power analysis”.

## Data Availability

The data that support the findings of this study are available on request from the corresponding author. The data are not publicly available due to privacy or ethical restrictions.
